# Strategies against Nonsense: Oxadiazoles as Translational Readthrough-Inducing Drugs (TRIDs)

**DOI:** 10.3390/ijms20133329

**Published:** 2019-07-06

**Authors:** Ambra Campofelice, Laura Lentini, Aldo Di Leonardo, Raffaella Melfi, Marco Tutone, Andrea Pace, Ivana Pibiri

**Affiliations:** Dipartimento di Scienze e Tecnologie Biologiche, Chimiche e Farmaceutiche (STEBICEF), Università Degli Studi di Palermo, Viale delle Scienze Ed. 16-17, 90128 Palermo, Italy

**Keywords:** premature termination codon, nonsense mutation, translational readthrough inducing drugs, ataluren, oxadiazoles, cystic fibrosis

## Abstract

This review focuses on the use of oxadiazoles as translational readthrough-inducing drugs (TRIDs) to rescue the functional full-length protein expression in mendelian genetic diseases caused by nonsense mutations. These mutations in specific genes generate premature termination codons (PTCs) responsible for the translation of truncated proteins. After a brief introduction on nonsense mutations and their pathological effects, the features of various classes of TRIDs will be described discussing differences or similarities in their mechanisms of action. Strategies to correct the PTCs will be presented, particularly focusing on a new class of Ataluren-like oxadiazole derivatives in comparison to aminoglycosides. Additionally, recent results on the efficiency of new candidate TRIDs in restoring the production of the cystic fibrosis transmembrane regulator (CFTR) protein will be presented. Finally, a prospectus on complementary strategies to enhance the effect of TRIDs will be illustrated together with a conclusive paragraph about perspectives, opportunities, and caveats in developing small molecules as TRIDs.

## 1. Introduction

**Figure 1:**
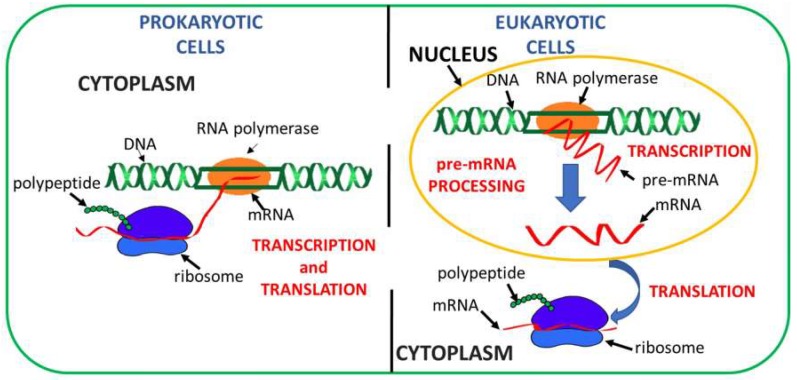
Steps involved in protein synthesis in prokaryotic (**left**) and eukaryotic cells (**right**).

Protein synthesis is a crucial phase for any living organism; therefore, any phenomenon affecting any of the steps involved in such a crucial process could jeopardize life itself or cause severe disorders. Protein synthesis, in the classical vision of the central dogma, consists of two steps: transcription and translation (Figure 1) [1,2,3]. Therefore, any strategy aiming at correcting miscarried protein syntheses should intervene before the synthetic process is completed. In eukaryotic cells, as opposed to prokaryotic cells (Figure 1, left), transcription and translation are spatially and temporally separated, since transcription, which is followed by pre-mRNA processing, takes place in the nucleus, while translation occurs in the cytoplasm (Figure 1, right). The mature mRNA exits the nucleus, reaches the cytoplasm, where the ribosome ties itself to the mRNA’s start codon, and begins the translation process to produce a peptide chain. Protein synthesis goes on until the ribosome meets a stop triplet (UGA, UAA, or UAG), thus ending translation and releasing the synthesized polypeptide [4].

In this context, mutations are alterations in the DNA sequence that are reflected in the gene transcript and, when nonconservative, can result in the production of nonfunctional proteins or even hinder protein expression. Among all types of mutations, base substitutions (also known as point mutations) are the most common and can belong to the classes of silent, missense, or nonsense mutations [5]. Silent mutations are harmless since they change a codon for a different one that codes for the same amino acid, thus resulting in the production of a protein identical to the wild-type one. Missense mutations, instead, lead to a codon that would be translated into a different amino acid with respect to the wild type; this mutation would have deleterious effects on the functionality of the resulting protein only when the new amino acid has different properties with respect to the one coded in the wild-type gene. Finally, nonsense mutations are the most dangerous point mutations. In fact, when UGA, UAA, and UAG codons appear earlier in the mRNA sequence, with respect to the normal stop signal, nonsense mutations are interpreted as a premature termination codon (PTC). Once the ribosome encounters a PTC in its acceptor (A) site, translation will be terminated, producing then a truncated and nonfunctional protein. This could lead to severe forms of various diseases, unless the truncation occurs in nonfunctional terminal parts of the wild-type protein [6]. Since truncated polypeptides synthesized from these aberrant transcripts could be toxic to normal cellular functions, mammalian cells evolved a mechanism to monitor the quality of mRNAs. Thus, the transcripts harboring a PTC are subject to degradation by the nonsense-mediated mRNA decay (NMD) pathway [7].

Unfortunately, nonsense mutations are the cause of about 11% of all genetic disorders in humans [8,9]. In particular, it was observed that stop mutations are associated to disorders such as cystic fibrosis (CF) [6], Duchenne muscular dystrophy (DMD) [10], spinal muscular atrophy [11,12], neurofibromatosis [13], retinitis pigmentosa [14,15], lysosomal storage disease [16], ataxia telangiectasia (AT) [17], Hurler’s syndrome (HS) [6], Rett syndrome [18], Shwachman–Diamond syndrome [19], Usher’s syndrome (USH) [20], Hemophilia A and B [21], Tay–Sachs disease [22], and several forms of cancer [23].

Therefore, exploring novel therapeutic approaches for the treatment of such a wide range of diseases caused by PTCs would make a significant contribution to improve the health conditions of many patients [24]. While some therapies aim at diminishing the impact of symptoms of these diseases, recent research is facing the challenge of targeting the genetic defect itself, in the frame of a personalized medicine approach. On one hand, correction of the genetic defect at the DNA level has been attempted by genome editing strategies in order to override nonsense mutations during transcription [25,26,27,28]. On the other hand, drugs have been developed to target the translation phase and promote the bypass of the PTC, allowing the synthesis of full-length functional proteins, a strategy known as PTC “readthrough” [9,24,29,30]. This review illustrates the different classes of drugs that have been developed so far, in order to promote the readthrough of premature stop codons, focusing particularly on oxadiazole derivatives, their proposed mechanism of action, and recent results in restoring the production of the full-length cystic fibrosis transmembrane regulator (CFTR) protein.

## 2. Concepts behind Readthrough Approaches

**Figure 2:**
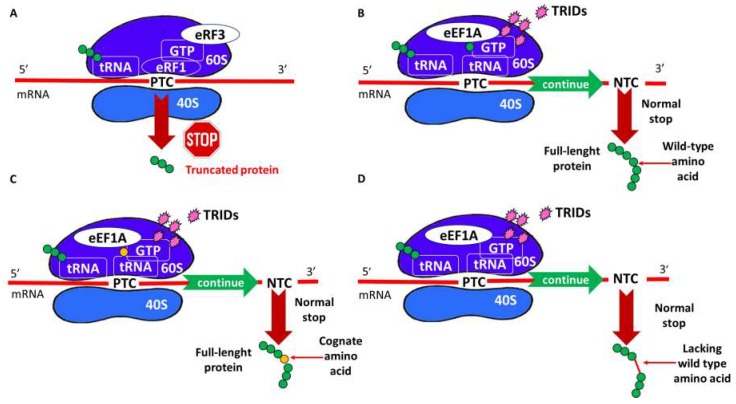
(**A**) Translation of premature termination codon (PTC)-containing mRNA in the absence of translational readthrough-inducing drugs (TRIDs); (**B**) ideal readthrough of PTC; (**C**) readthrough inserting a cognate amino acid; and (**D**) readthrough skipping amino acid insertion. Figure legend: mRNA, RNA messenger; tRNA, RNA transfer; 40S and 60S, small and large ribosome subunit, respectively; eEF1A, eukaryotic translation elongation factor 1A; eRF1 and eRF3, eukaryotic translation termination factor 1 and 3, respectively; and GTP, guanosine-5’-triphosphate.

Since nonsense mutations alter polypeptide synthesis, eventually leading to truncated proteins (Figure 2A), the main goal of readthrough approaches is to restore the production of a functional protein that, even in small amounts, can diminish the symptoms of nonsense disorders. Considering that protein synthesis is carried out on ribosomes, the latter is the logical place where the desired correction should occur. In this context, different small molecules have been developed as nonsense suppressors (NonSups), or translational readthrough-inducing drugs (TRIDs) [31], to fool the ribosomal machinery during recognition of the PTC as a stop signal, thus avoiding the premature ending of protein synthesis [9]. However, because there are a number of actors (mRNA, tRNA, release factors, ribosomal subunits, etc.) involved in peptide synthesis, TRIDs can affect various biomolecular targets, leading to different types of readthrough (Figure 2B–D). The ideal readthrough process (Figure 2B) should be highly specific and rely on: (i) a sufficient level of mRNA that survived NMD degradation to restore enough functional full-length protein; (ii) insertion of the correct amino acid (the same as that coded by the wild-type gene) at the point of the sequence that would have been interrupted by the premature stop; and (iii) recognition of the normal termination codon (NTC in Figure 2) that leads to the correct termination of protein synthesis. Therefore, ideal TRIDs should be able to distinguish between normal and premature termination codons to achieve the synthesis of a functional full-length protein. However, if TRIDs induce the insertion of a different amino acid (with respect to the wild type) (Figure 2C) or just skip the PTC, continuing peptide synthesis without inserting any amino acid in the place of the PTC (Figure 2D), this could cause a misfunction of the resulting protein [22]. This could lead, for instance, to uncorrected folding, hindering the subcellular localization of the protein in its normal site of action or, in case of a channel protein, to narrower channels that inhibit the normal flow of ions. Moreover, if TRIDs act also as (or are used in conjunction with) NMD inhibitors to implement the RNA levels to readthrough, it must be considered that interaction with the NMD mechanism could be a problem for the cells, leading to the survival of other mutant mRNAs that should be degraded.

In this context, the first class of small molecules that showed activity as TRIDs was that of aminoglycoside antibiotics [32], later followed by the discovery of Ataluren [33] and its oxadiazole analogues [29,34,35]. These have been tested both in vitro and in vivo, observing their interaction with biomolecules involved in protein synthesis or their interference with the mechanism of NMD [9]. However, the detailed mechanism of action of these new readthrough inducers is still uncertain, and this is the main reason for current limited use of TRIDs for human therapy. The following paragraphs describe the activity of various classes of TRIDs, comparing hypotheses and data on their mechanism of action.

## 3. Classes of Translational Readthrough-Inducing Drugs (TRIDs)

### 3.1. Aminoglycosides

**Figure 3:**
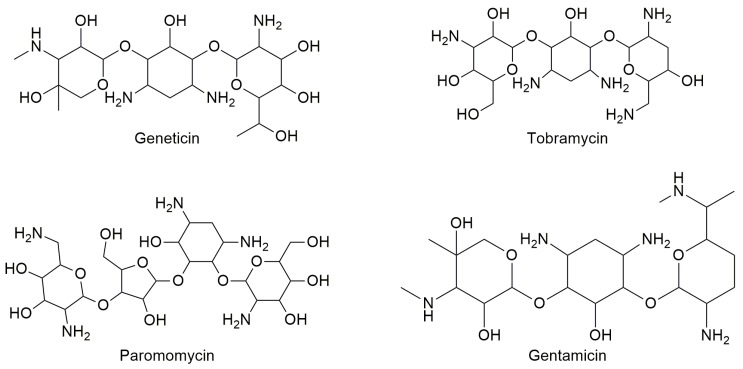
Structures of some aminoglycoside antibiotics studied for readthrough ability.

Aminoglycosides are derivatives of polyamino oligosaccharides commonly used as antibiotics to treat Gram-negative antibacterial infections [36]. In fact, they are capable of binding to the site of the bacterial small ribosome unit that generally controls codon–anticodon interactions between mRNA and tRNA [37]. Considering their ribosomal activity, compounds such as gentamicin, tobramycin, paromomycin, and geneticin (Figure 3) were also studied for their readthrough-inducing features.

**Figure 4:**
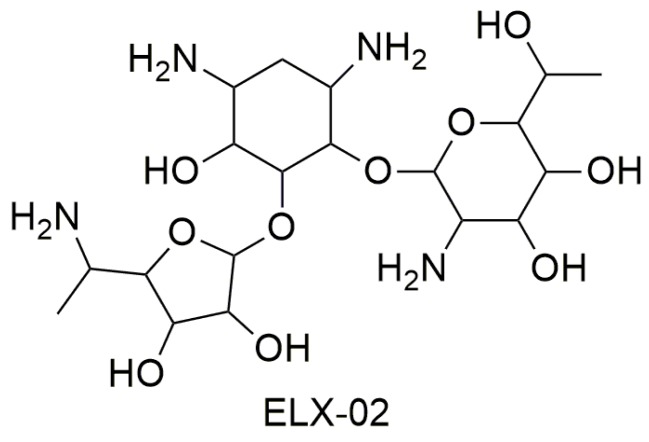
Structure of ELX-02.

Most studies have demonstrated that tobramycin has low efficiency as a PTC suppressor, while gentamicin is very efficient as a TRID although it presents an undesirable toxic profile because of its ototoxicity and nephrotoxicity [38]. Some studies have demonstrated that the activity of aminoglycoside TRIDs varies depending on the stop codon sequence (UGA, UAA, or UAG) [32,39,40]. Moreover, the negative side effects of this class of drugs as TRIDs are also due to the readthrough of correctly positioned stop codons, thus demonstrating a lack of selectivity towards the premature stop codon. Additionally, aminoglycoside antibiotics cannot be used for long-term therapy (as it would be required by their use as TRIDs) because of the risk of inducing bacterial resistance [37]. An interesting alternative to gentamicin is represented by ELX-02 (Figure 4), a synthetic aminoglycoside that binds eukaryotic ribosomes and has been recently claimed to induce the readthrough of PTCs without the toxicity of the antibiotics.

The first study showed that ELX-02 is capable both to restore the synthesis of the CFTR protein in the presence of nonsense mutations and to increase the levels of mRNA, which indicates that the drug is capable to interact with the NMD mechanism and/or to stabilize the transcript [37,41].

ELX-02 exercises its readthrough activity by stabilizing the “exo” conformation of two adenosine residues in rRNA. Because of this conformation, a near-cognate tRNA can bind the PTC, shifting aside eRF1. In this way, protein synthesis will continue beyond the PTC in order to generate a full-length protein. ELX-02 shows greater selectivity for cytoplasmic ribosomes than gentamicin, which results in increased readthrough activity and lower toxicity. In fact, aminoglycoside toxicity is determined by its affinity for mitochondrial ribosomes, and ELX-02’s affinity for this site is 100-fold lower than other aminoglycoside antibiotics. As for clinical studies, ELX-02 underwent randomized double-blind placebo-controlled trials. It was administered subcutaneously in doses of 0.3 to 7.5 mg/kg and intravenously in doses of 0.3 mg/kg. These studies confirmed that ELX-02 administered subcutaneously is widely bioavailable, well-tolerated, and does not present toxic effects, such as nephrotoxicity and ototoxicity, when administered in doses within the therapeutic range [37].

### 3.2. Oxadiazoles

**Figure 5:**
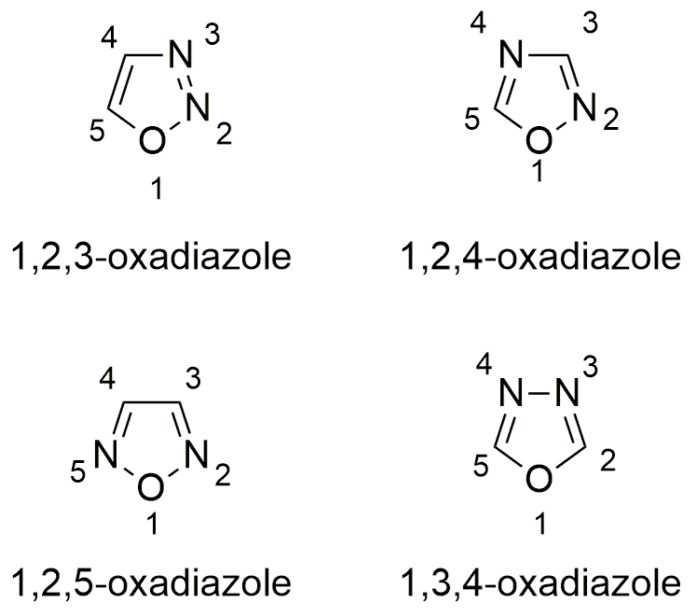
Structures of oxadiazoles.

Oxadiazoles are aromatic heterocyclic rings containing one oxygen and two nitrogen atoms occupying different ring positions as in 1,2,3- [42], 1,2,4- [43,44], 1,2,5- [45], and 1,3,4-oxadiazoles [46,47] (Figure 5). Depending on the relative position of nitrogen and oxygen atoms, these classes of oxadiazoles possess different characteristics. For instance, 1,2,4-oxadiazoles have lower water solubility than 1,3,4-oxadiazoles because of the reduced hydrogen bond acceptor character of the nitrogen in 1,2,4-oxadiazole ring [43]. Moreover, they have been extensively studied because of their peculiar reactivity [43,48] and possible applications in material chemistry [49,50,51,52,53,54,55] or as bioactive compounds [44,56,57,58].

Of all the four possible classes of oxadiazoles, 1,2,4- and 1,3,4-oxadiazoles are the most studied for their pharmaceutical properties [59]. Their heterocyclic core is present in commercial drugs, such as the antitussive oxolamine (Perebron^®^) [60] and antiviral (Raltegravir^®^) [61], or potential anticancer drug candidates [62].

**Figure 6:**
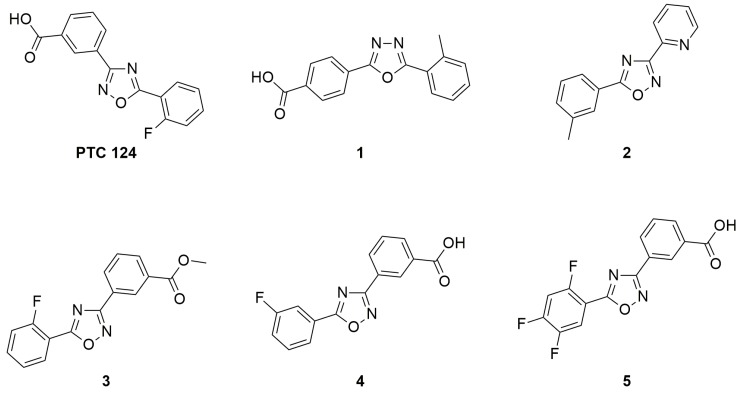
Structure of Ataluren (also known as PTC124 or Translarna^®^) and its most active analogues.

In 2007, a fluorinated 1,2,4-oxadiazole, specifically the 3-[5-(2-fluorophenyl)-1,2,4-oxadiazol-3-yl]-benzoic acid also known as Ataluren or PTC124 (Figure 6), was reported to promote the readthrough of nonsense mutations [33]. Ataluren is capable of promoting the readthrough of UGA, UAG, and UAA codons, but it shows the highest readthrough activity for the UGA codon [6,33]. Since Ataluren is structurally different from aminoglycosides, its discovery opened the way to a different class of drugs able to treat genetic disorders caused by nonsense mutations. Additionally, its activity profile within a 0.01–3 µM therapeutic range was better than that of gentamicin aminoglycoside, which was active as a TRID at much higher concentrations [6,33]. Furthermore, Ataluren is able to distinguish between normal and premature stop codons, thus inducing selective readthrough of PTCs without showing the typical toxicity of aminoglycosides. Indeed, several clinical trials have emphasized the safety and tolerability of Ataluren, even for long-term treatments [6,33,63].

**Figure 7:**
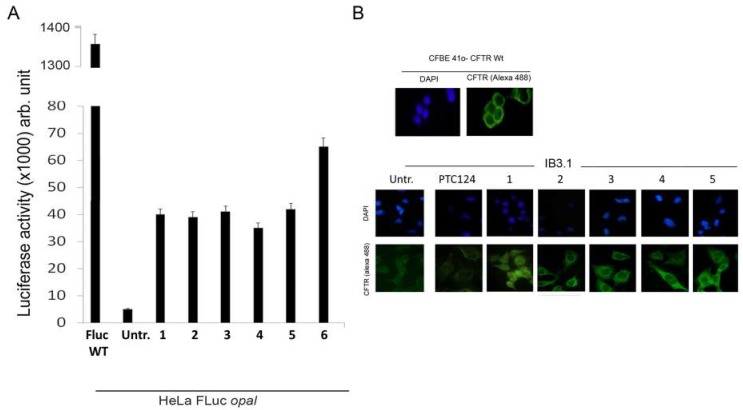
(**A**) Histogram of luciferase activity shown by HeLa cells after treatment with some of the most active compounds compared with untreated cells and with Fluc used as positive control; (**B**) Immunofluorescence of IB3.1 cells untreated (Untr; negative control) or treated with PTC124 (Ataluren; positive control) and compounds 1–5 respectively. Cystic fibrosis transmembrane regulator (CFTR) protein was revealed by a specific antibody targeting its first external loop (secondary antibody in green, Alexa-488). Nuclei (blue) were DAPI (4′,6-diamidino-2-phenylindole) stained.

Today, Ataluren is marketed under the trade name of Translarna^®^ and has been approved as a therapy for ambulatory patients aged five years and older affected by Duchenne muscular dystrophy [64]. However, its use is not generally extensible to a wide range of cases since not all patients suffering from pathologies caused by nonsense mutations found beneficial effects, especially if Ataluren is administered in conjunction with other drugs [65]. For instance, in an Ataluren clinical trial performed on patients affected by cystic fibrosis, and taking tobramycin by inhalation, the competitive action between Ataluren and aminoglycoside antibiotics was highlighted [65]. This competition could be due to the fact that Ataluren also targets the ribosomal machinery as well as tobramycin, although the actual biomolecular target could be different for the two drugs. Indeed, Ataluren’s readthrough activity has been questioned [66,67,68], and its detailed mechanism of action is still debated, despite the fact that the functionality of rescued full-length proteins has been demonstrated [69,70] and consensus is converging towards its interaction with mRNA [71]. Confirmation of Ataluren’s readthrough activity by orthogonal in vitro assays [72] prompted researchers to optimize this lead compound by designing Ataluren’s analogues that could be used for the treatment of diseases caused by nonsense mutations. The modification of Ataluren’s scaffold could involve either the oxadiazole heterocyclic core or the attached lateral moieties. In this context, and in the absence of a certain biomolecular target, computational optimization of Ataluren’s analogues could be achieved by ligand-based virtual screening. Thus, virtually selected sets of molecules containing either the 1,2,4- or the 1,3,4-oxadiazole core were synthesized and experimentally tested by FLuc cell-based assays to evaluate their readthrough ability [29,34,35,72]. In particular, Hela cells were transfected with pFLuc wild-type (pFLuc-wt) or pFLuc-opal (containing the UGA stop codon) plasmids. The expression of *FLuc* gene was then evaluated by measuring the luminescence of the produced luciferase in cells treated with the tested derivatives. However, Auld et al. [67] questioned the validity of FLuc cell-based assays as a method to evaluate the readthrough activity of Ataluren and its analogues, claiming that these compounds stabilized FLuc, protecting it from its degradation by trypsin. Therefore, an orthogonal assay was developed to confirm the readthrough activity of the new compounds that were the most active according to the Fluc assay (Figure 7A).

To this aim, a reporter plasmid (H2B-GFP-opal), in which the cDNA of the *H2B* gene is fused to the cDNA of the green fluorescent protein (GFP) harboring a PTC at Trp58 [72], has been generated. Hela cells were then transfected with the normal (H2B-GFP) and the mutated (H2B-GFP-opal) plasmids and treated with selected compounds. The detection of H2BGFP by immunofluorescence demonstrated the synthesis of a full-length H2BGFP protein and hence the occurrence of readthrough. These results were confirmed also by western blotting, indicating the achievement of a full-length protein [29,34,35]. The most active compounds were also tested for nonsense suppression in the cystic fibrosis bronchial epithelial cell line IB3.1 derived from a CF patient. Also in this case, fluorescence microscopy demonstrated the readthrough capacity of these compounds with the consequent restoring of full-length CFTR and its correct localization on the cell membrane (Figure 7B). Moreover, western blotting was used to quantify the CFTR level, and it confirmed the significant increase in protein level in cells treated with Ataluren analogues compared to untreated cells [29,34,35].

Once the readthrough activity of Ataluren-like compounds was also confirmed on the CF model, leading to a significant increase of CFTR expression (compared to untreated cells) and to its correct location on the cell membrane, the physiological functionality of the recovered protein needed to be assessed. For this purpose, Fisher rat thyroid (FRT) cells transfected with a plasmid vector harboring the mutated (G542X) CFTR were used to evaluate expression and activity of the CFTR channel. Fluorescence microscopy images indicated the readthrough of the PTC and the correct position of CFTR at the cell membrane; western blotting allowed to quantify the level of CFTR protein. The functionality of the CFTR channel can be evaluated by two approaches: on one hand, FRT cells capable of differentiating in an epithelium can be used to measure the ion current of chloride ions crossing the epithelium in an Ussing flux chamber, thus giving a functionality response [73]. On the other hand, FRT cells expressing EYFP protein (an ectopically expressed mutant form of the yellow fluorescent protein) can be used for a quench-EYFP assay based on iodide-mediated fluorescence quenching to evaluate the halide ion flow across the cell membrane [74]. With both approaches an increase in the functionality of the chloride channel was observed when nonsense CFTR^(G542X-opal)^ FRT cells were treated with compound one, demonstrating its activity as a TRID [35].

### 3.3. Miscellanea

**Figure 8:**
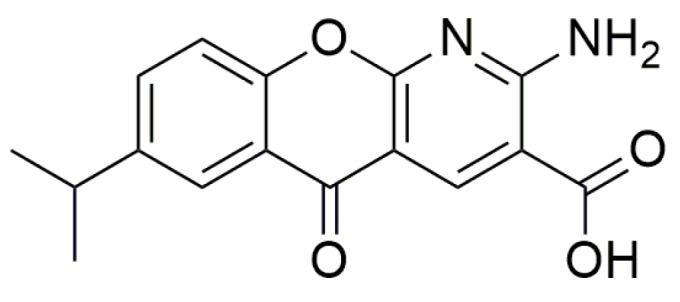
Structure of Amlexanox.

Besides oxadiazoles and aminoglycosides, other small molecules have recently been tested as TRIDs to treat genetic disorders caused by nonsense mutations, and results concerning their activity are summarized below. Amlexanox (Figure 8), an antiallergic and anti-inflammatory agent that is administered orally or topically to treat asthma and aphthous ulcers [75,76,77], was discovered to inhibit NMD and induce PTC readthrough. Although this double action has been previously described for geneticin as well as for other TRIDs, Amlexanox is less toxic and active at lower concentrations than those required for geneticin. Additionally, apparently Amlexanox is somehow specific and does not interfere with naturally occurring NMD processes. In this way, the increased number of mRNAs surviving the NMD results in some recovery of a full-length protein [75].

**Figure 9:**
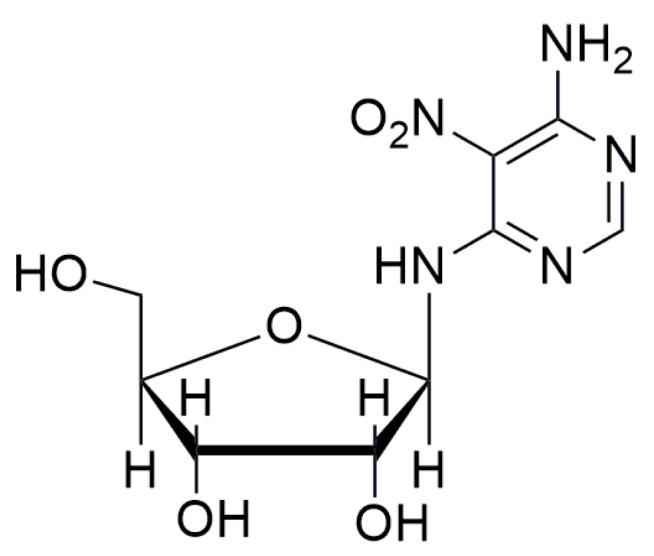
Structure of Clitocine.

Recently, the nucleoside analogue Clitocine (Figure 9) was discovered to induce readthrough with a TRID activity two to three orders of magnitude higher than gentamicin and geneticin [78]. Furthermore, Clitocine has a greater selectivity towards PTCs than towards normal stop codons. Interestingly, to exert its readthrough activity, it is necessary that Clitocine is incorporated into mRNA after its conversion into Clitocine-5′-triphosphate. Friesen et al. [78] claim that, surprisingly, the incorporation of Clitocine in the mRNA sequence increased the readthrough of PTCs, and that readthrough activity was dose-dependent. Moreover, this nucleoside analogue is able to induce the readthrough in tumors harboring a nonsense mutation onto the *p53* gene, thus inhibiting the growth of tumors.

Other approaches to suppress the effect of nonsense mutations involve genetic engineering and include the administration of CRISPR-Cas9 (Crispr-ASsociated), a nuclease that cuts DNA in a sequence-specific manner guided by a complementary sequence of RNA (gRNA). This strategy, tested for the correction of the CFTR gene, did not lead to sufficient repair and introduced issues concerning the use of appropriate vectors for the administration of the complex [25]. On the other hand, a recent study reported the use of anticodon engineered transfer RNAs (ACE-tRNA) as a possible strategy to suppress PTCs and introduced a near-cognate amino acid in the growing peptide chain. Since ACE-tRNA showed limited interactions with normal stop codons, the use of these engineered biomolecules represents a promising approach for selective readthrough [79].

## 4. TRID Mechanism of Action

Considering the factors participating in the translation of protein synthesis, TRIDs can interact with various biologic targets, affecting the phase in which they are involved and leading to the readthrough of a PTC. In principle, these targets could be: (i) release factors (RFs), to hinder their interaction with the stop codon; (ii) ribosomal RNA, to affect the context where PTC/RF or codon/anticodon recognition occurs; and (iii) messenger or transfer RNA to affect codon/anticodon recognition, either from the PTC or the anticodon side, thus inducing PTC/anticodon recognition. In the absence of a translational readthrough inducer, the readthrough of a premature termination codon can occur in about 1% of cases (tenfold more frequently than readthroughs of normal stop codons) through normal competition between tRNA and RF for entering the ribosome A-site [22].

Because of their known antibiotic activity, aminoglycosides have been thoroughly studied for their interaction with the ribosome. Additionally, crystal structures are available that demonstrate the specific binding site of representative aminoglycosides such as paromomycin [22,80] or geneticin (G418) [22,81]. 

On these bases, consensus has been reached upon the readthrough mechanism of action of aminoglycosides supported by the comparison of different crystal structures of ribosome/aminoglycoside and ribosome/RF complexes. In particular, crystal structures demonstrated that binding of aminoglycosides produces a conformational change in the ribosomal A-site with flipping of two adenine residues of the rRNA [22,80,81]. Superimposition of this conformationally changed structure with the crystal structure of the ribosome/RF complex suggests that, in the presence of aminoglycosides, the RF/ribosome interaction would be encumbered by steric hindrance [22]. Reasonably, although crystal structures of ribosome/ELX-02 have not been recorded, a similar mechanism has been reported for the readthrough activity of ELX-02 [37].

On the other hand, the structures of oxadiazole TRIDs are so different from those of aminoglycosides that analogies in their molecular mechanism of action appear unlikely.

Indeed, the molecular mechanism of action of Ataluren, the benchmark oxadiazole TRID, still demands unequivocal 3D experimental evidence [66]. In fact, recent discussion proposed that Ataluren binds to the ribosome A-site on the basis of its decreased activity in the presence of tobramycin [66,70]. Additionally, observed bias in Ataluren-induced tRNA mispairing leading to readthrough, compared to endogenous mispairing typical of basal readthrough, demonstrated that Ataluren can be selective towards the insertion of a near-cognate tRNA [70]. However, while these findings are undoubtedly the (direct or indirect) effect of some interaction between Ataluren and translational processes occurring in the ribosome, evidence is still lacking about the actual binding of Ataluren on a well-determined biomolecular target.

In this context and in the lack of crystal data, computational tools are crucial resources to compare the affinities between structurally-diverse drug candidates and their potential biomolecular targets.

A previous molecular dynamics study simulated the potential interaction between Ataluren and different types of PTCs centered on a 33-nucleotide mRNA fragment of the *CFTR* gene [72]. This study highlighted a more stable interaction with premature UGA codons with respect to UAG and UAA, in agreement with the experimentally observed higher suppression of opal versus amber and ochre nonsense mutations [33,72]. The driving forces that stabilize Ataluren/PTC complexes involve π–π stacking, H-bonding, and hydrophobic van der Waals interactions [72]. Interestingly, a readthrough activity comparable with that of Ataluren was also observed in its analogues devoid of fluorine or carboxylic moieties, suggesting that the oxadiazole core itself plays a crucial role as a pharmacophore [34].

Therefore, Ataluren’s mechanism of action has been further studied through complementary computational approaches, such as induced fit docking (IFD), quantum polarized ligand docking (QPLD), and molecular mechanics combined with the generalized Born and surface area continuum solvation (MM-GBSA) methods, and results were compared with those obtained for a series of oxadiazole analogues of Ataluren as well as with a series of aminoglycosides [71]. This study represented the first computational peer comparison of the strength of potential interactions between each TRID and different biomolecular targets involved in protein synthesis: the 16S and 18S subunits of ribosomal RNA, respectively, for bacteria and eukaryotes, the release factor eRF1, and mRNA [71]. Interestingly, both the positioning and the binding energy values associated with each TRID/target interaction showed that Ataluren/eRF1 interactions were not stable and could not be claimed as a cause for Ataluren-induced readthrough. Moreover, Ataluren and its analogues showed a tenfold weaker interaction with the ribosomal RNA compared to the rRNA/aminoglycoside interaction. On the other hand, the calculated binding energies for mRNA/Ataluren (or its analogues) interactions showed a marked preference for the UGA PTC as a target binding site [71,72]. This binding is further stabilized by π–π interactions between the aromatic portions of Ataluren and guanine, present at position –6 with respect to the UGA PTC, and by hydrogen bonds with uracil, cytosine, and guanine at positions –4, –3, and +1, respectively. Among these ancillary interactions, the one involving guanine in the –6 position is the most important to stabilize the binding to the UGA, as demonstrated by a virtual mutagenesis study that compared binding energies as a function of the genetic context surrounding the premature termination codon [71].

## 5. Increasing TRID Efficiency

### 5.1. Nonsense-Mediated mRNA Decay (NMD) Inhibition

Nonsense suppression drugs, hence, reduce the efficiency of translation termination at in-frame premature termination codons, thereby allowing ribosomes to resume translation elongation and generate a full-length protein. However, as already highlighted, the efficiency of readthrough can be limited by nonsense-mediated mRNA decay (NMD) [82].

Restoring the expression of nonsense-mutated genes by inhibition of the nonsense-mediated decay of transcribed mRNA, thus relying on basal readthrough for the production of a full-length functional protein, would be too limited to be effective as a therapeutic strategy.

**Figure 10:**
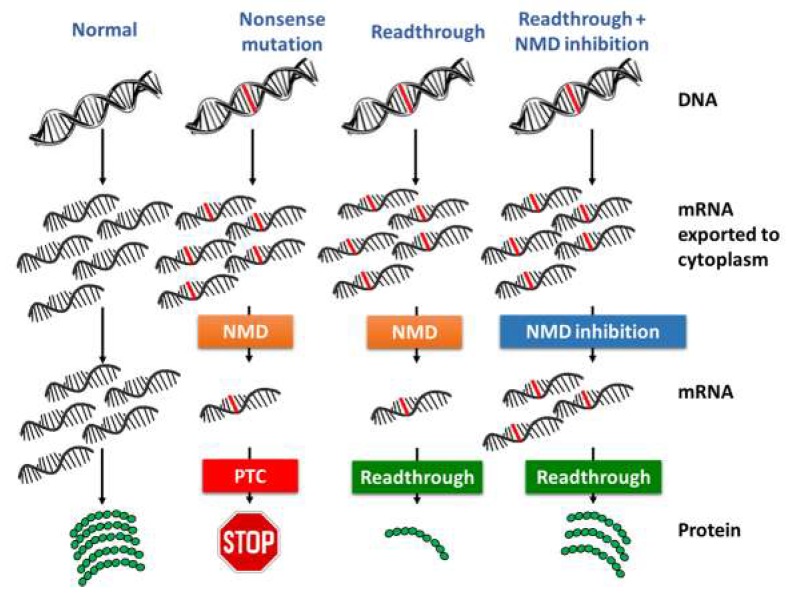
Comparison between protein syntheses from: a normally coded gene (**left**), a nonsense-mutated gene through normal NMD and codon recognition (**center-left**), a nonsense-mutated gene through normal NMD and active readthrough (**center-right**), and a nonsense-mutated gene with inhibited NMD and active readthrough (**right**).

The use of NMD inhibitors in conjunction with TRIDs would allow to overcome this issue and to increase the quantity of functional full-length protein [83]. This strategy, which is illustrated in Figure 10, has the main drawback of potentially inducing the production of, even toxic, mutated proteins, whose synthesis would have been blocked by the NMD surveillance mechanism [30].

Several drugs such as caffeine and SMG1 kinase inhibitors have been found to attenuate the NMD pathway by inhibiting Upf1 and Upf1 protein phosphorylation [84]. Inhibition of NMD may enhance the effect of nonsense suppression drugs to restore protein function by increasing steady-state mRNA abundance [84].

Some experiments evidenced that attenuation of the NMD pathway strategy to enhance the translation of full-length proteins could be a very good approach, although it probably requires further improvement in terms of nonsense mRNA target recovery [84,85]. This approach was recently tested by treating IB3.1 cells (a cystic fibrosis cell model harboring the W1282X stop mutation) with caffeine to increase levels of nonsense CFTR-mRNA to restore a functional protein produced by PTC suppression with Ataluren, thus providing a greater therapeutic benefit [86].

### 5.2. Correctors and Potentiators

The ideal readthrough process would result in the production of a fully functional protein; therefore, the continuation of protein synthesis beyond the PTC is only the first (necessary) step for this achievement. In fact, protein functionality also depends on its correct folding, a process that occurs after the completion of the polypeptide chain. In this context, drugs could be used to correct the protein conformation if the readthrough, with the introduction of a near-cognate amino acid, causes an imperfect folding of the protein.

**Figure 11:**
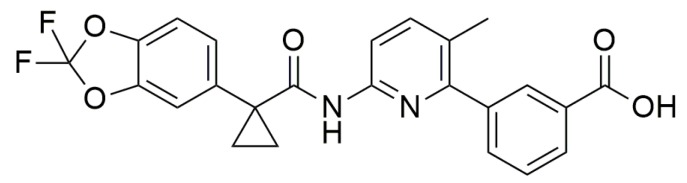
Structure of Lumacaftor.

Examples of this approach presented in this paragraph would focus only on the cystic fibrosis transmembrane regulator (CFTR) protein. For instance, “CFTR correctors” can act with two mechanisms: binding CFTR protein and promoting its correct folding and maturation, such as Lumacaftor (Figure 11), or modulating some phases of protein regulation [25].

**Figure 12:**
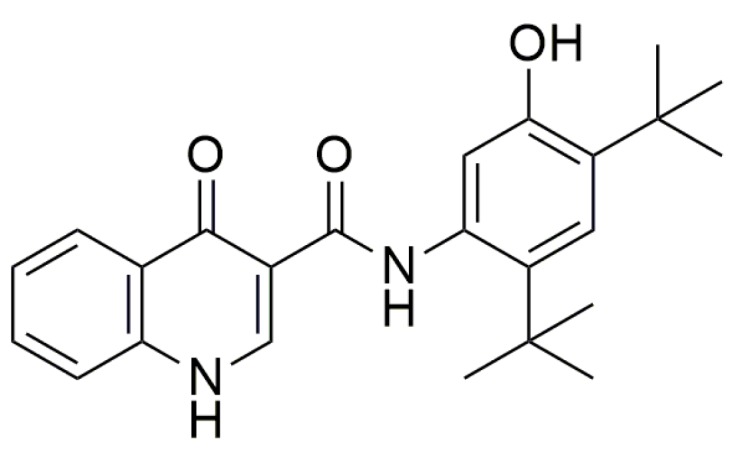
Structure of Ivacaftor.

Alternatively, “CFTR potentiators” are small molecules that increase the possibility of CFTR’s opening in the presence of an agonist. These drugs can be used in the case of patients that have gating problems. Ivacaftor (Figure 12) was approved by FDA in patients, aged six and above, that have CFTR gating mutations; it acts by increasing chloride levels in the presence of ΔF508 [25].

Combined use of both potentiators and correctors has also been proposed, although the costs of these types of therapies have been estimated above 300,000 USD per year. These functions could be joined in dual-acting molecules that are cleaved by intestinal hydrolytic enzymes producing both the CFTR corrector and the CFTR potentiator [25].

## 6. Conclusions and Perspectives

Nonsense mutations are the cause of numerous genetic diseases for which a definite therapy is still far from being reached. Synergies between chemists, biologists, and physicians are essential to approach the problem through a holistic perspective. This approach should apply not only to the improvement of drugs able to induce the readthrough of premature termination codons but also to the detailed understanding of their mechanism of action. In fact, in this particular research field, details of molecular drug–target interactions would hopefully explain discrepancies in the observed activity of drug candidates or even suggest further directions for TRID development. Indeed, efficacy of the readthrough process could depend on the identity of the premature stop codon as well as on the context surrounding it. For this reason, a careful screening of patients’ gene sequence, together with in vitro readthrough activity data collected on patients’ isolated cells, will be relevant to confirm such hypotheses. Moreover, the readthrough drug should not interfere with the recognition of normally positioned stop codons, thus allowing for the correct termination of protein synthesis. Finally, recovered polypeptides should preserve the functionality of the wild-type protein. Several solutions have been proposed to tackle this challenge, by means of small molecules (e.g., aminoglycosides and oxadiazoles) or genetic engineering (e.g., CRISPR-Cas and ACE-tRNAs) approaches. Aminoglycoside antibiotics are able to induce the readthrough of premature stop codons by interacting with the small unit of the ribosome. However, their lack of selectivity may cause the readthrough of normal stop codons and increase their toxicity, hindering their application for prolonged therapies. This issue might have been addressed by the discovery of ELX-02, a glycoside that has been reported both as a TRID and inhibitor of NMD, although structural details on its supposed interaction with the ribosome need further in-depth studies. 

On the other hand, oxadiazole TRIDs were more selective than aminoglycosides in inducing the readthrough of premature but not normal stop codons. Moreover, they did not show the toxicity of aminoglycosides and were active at lower dosages. However, no crystal structure is available to confirm their binding site on the biomolecular target. Interaction of oxadiazole TRIDs with mRNA has been proposed on the basis of computational studies showing differences with the other classes of TRIDs and highlighting stabilizing interactions involving the genomic context surrounding the PTC. 

As for genetic engineering approaches, the use of ACE-tRNA, based on anticodon recognition, could resolve selectivity issues towards the identity of the PTC, and selectivity towards premature vs. normal stop codon, but could raise issues of off-target activity. Moreover, at this stage, costs associated with this approach appear significantly higher if compared with those involved with the development of small TRIDs.

In summary, TRIDs currently represent the most promising strategy to achieve the synthesis of a full-length protein from nonsense-mutated genes. Although it is still possible that the produced protein is less functional with respect to the wild-type one, the use of TRIDs would allow to convert a serious genetic disorder in a milder genetic defect. In this context, further development could be envisaged by coupling the use of TRIDs with NMD inhibitors, protein folding correctors, or function enhancers.

## Abbreviations

ACE-tRNAs	Anticodon engineered transfer RNAs
AT	Ataxia telangiectasia
CF	Cystic fibrosis
CFTR	Cystic fibrosis transmembrane conductance regulator
DAPI	4′,6-diamidino-2-phenylindole
DMD	Duchenne muscular dystrophy
FTR cells	Fisher rat thyroid cells
GFP	Green Fluorescent Protein
HS	Hurler’s syndrome
IFD	Induced Fit Docking
MD	Molecular Dynamics
MM-GBSA	molecular mechanics /generalized Born and surface area continuum solvation
NMD	Nonsense-mediated mRNA decay
NonSups	Nonsense suppressors
pFLuc-wt	Plasmid firefly luciferase-wild type
PTC	Premature stop codon
PTC124	Ataluren or 3-[5-(2-fluorophenyl)-1,2,4-oxadiazol-3-yl] benzoic acid
QPLD	Quantum Polarized Ligand Docking
RF	Release factor
RLuc	*Renilla reniformis* luciferase
TRIDs	Translational Readthrough-Inducing Drugs
Upf1	Regulator of nonsense transcripts 1
USH	Usher’s syndrome
YFP	Yellow fluorescent protein
